# Microtubule‐assisted mechanism for toxisome assembly in *Fusarium graminearum*

**DOI:** 10.1111/mpp.13015

**Published:** 2020-11-17

**Authors:** Zehua Zhou, Yabing Duan, Jie Zhang, Fei Lu, Yuanye Zhu, Won Bo Shim, Mingguo Zhou

**Affiliations:** ^1^ College of Plant Protection Nanjing Agricultural University Nanjing China; ^2^ The Key Laboratory of Plant Immunity Nanjing Agricultural University Nanjing China; ^3^ Department of Plant Pathology and Microbiology Texas A&M University College Station Texas USA

**Keywords:** ɑ_1_–β_2_ tubulin heterodimer, carbendazim, DON, *Fusarium graminearum*, microtubule, toxisome

## Abstract

In *Fusarium graminearum*, a trichothecene biosynthetic complex known as the toxisome forms ovoid and spherical structures in the remodelled endoplasmic reticulum (ER) under mycotoxin‐inducing conditions. Previous studies also demonstrated that disruption of actin and tubulin results in a significant decrease in deoxynivalenol (DON) biosynthesis in *F. graminearum*. However, the functional association between the toxisome and microtubule components has not been clearly defined. In this study we tested the hypothesis that the microtubule network provides key support for toxisome assembly and thus facilitates DON biosynthesis. Through fluorescent live cell imaging, knockout mutant generation, and protein–protein interaction assays, we determined that two of the four *F. graminearum* tubulins, α_1_ and β_2_ tubulins, are indispensable for DON production. We also showed that these two tubulins are directly associated. When the α_1_–β_2_ tubulin heterodimer is disrupted, the metabolic activity of the toxisome is significantly suppressed, which leads to significant DON biosynthesis impairment. Similar phenotypic outcomes were shown when *F. graminearum* wild type was treated with carbendazim, a fungicide that binds to microtubules and disrupts spindle formation. Based on our results, we propose a model where α_1_–β_2_ tubulin heterodimer serves as the scaffold for functional toxisome assembly in *F. graminearum*.

## Graphical abstract

By serving as the scaffold, we propose a α_1_–β_2_ tubulin heterodimer‐assisted mechanism for functionally active toxisome assembly in *Fusarium graminearum*.

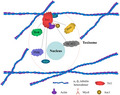

## 1 INTRODUCTION

*Fusarium graminearum* is the causal agent of fusarium head blight (FHB), which is a destructive disease of wheat and other small grain crops worldwide (Goswami & Kistler, 2004). In addition to decreasing crop yield and quality, the pathogen contaminates crops with trichothecenes, a group of mycotoxins with serious threats to human and animal health (Alexander et al., 2009; Goswami & Kistler, 2004). Mycotoxins such as deoxynivalenol (DON), nivalenol (NIV), and their acetylated derivatives (3‐ADON, 4‐ANIV, and 15‐ADON) all belong to type B trichothecenes, of which 3‐acetyldexynivalenol (3‐ADON) is the most frequently detected type in Asia (Alexander et al., 2011; Audenaert et al., 2013; Bennett & Klich, 2003). Notably, trichothecenes have been shown to be a vital virulence factor of *Fusarium* species (Alexander et al., 2009; Goswami & Kistler, 2004). Our previous study also showed that the inhibition of DON biosynthesis can lead to effective control of FHB (Li et al., 2019). In‐depth research into the biosynthesis and regulation of trichothecenes can provide crucial understanding for the development of novel control strategies, not only for *Fusarium*‐incited disease control but also for grain storage management.

**FIGURE 1:**
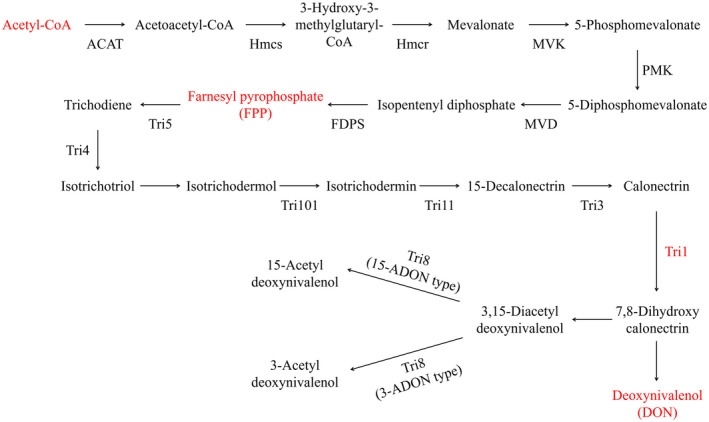
Deoxynivalenol (DON)biosynthetic pathway. The de novo DON biosynthetic pathway transforms acetyl‐CoA to DON in multiple steps. ACAT, acetyl‐CoA acetyltransferase (ACAT1, FGSG_05087; ACAT2, FGSG_09321); Hmcs, hydroxymethylglutaryl‐CoA synthase (FGSG_09266); Hmcr, 3‐hydroxy‐3‐methylglutaryl‐CoA reductase (FGSG_09197); MVK, mevalonate kinase (FGSG_05912); PMK, phosphomevalonate kinase (FGSG_09764); MDV, diphosphomevalonate decarboxylase (FGSG_10424); FDPS, farnesyl pyrophosphate synthetase (FGSG_06784); Tri5, trichodiene synthetase (sesquiterpene cyclase, FGSG_03537); Tri4, trichodiene oxygenase (cytochrome P450, FGSG_03535); Tri101, trichothecene 3‐O‐acetyltransferase (FGSG_07896); Tri11, isotrichodermin C‐15 hydroxylase (FGSG_03540); Tri3, trichothecene 15‐O‐acetyltransferase (FGSG_03534); Tri1, calonectrin oxygenase (FGSG_00071); Tri8, trichothecene 3‐O‐esterase (FGSG_03532). Hmcr, Tri4,and Tri1 are localized in the toxisome

The concept of metabolic channelling was first proposed by Srere more than 30 years ago (Srere, 1987). All cytoplasm contains various large molecules, such as proteins, nucleic acids, polysaccharides, even some toxic intermediaries or inhibitors. High concentrations of these molecules could influence many aspects of cellular function and metabolism (Ellis & Minton, 2003). Enzymes involved in the same biochemical pathway conventionally assemble to catalyse several specific consecutive reactions and these enzyme complexes are called metabolons (Clegg, 1984). Normally, the formation of metabolons is in response to a condition requiring adaption in metabolic flux, which results in enhanced conversion (Kohnhorst et al., 2017; Thomas et al., 2017). While these theories are now widely accepted, various underlying molecular mechanisms are still fascinating research topics. To date, a variety of examples in mammalian and human metabolons have been characterized in glycolysis, the tricarboxylic acid cycle, and many other metabolic pathways (An et al., 2010; Haanstra et al., 2016; Haggie & Verkman, 2002; Kohnhorst et al., 2017). In fungal species, there are few examples of characterized metabolons. In *Fusarium* species, enzymes involved in the de novo trichothecene biosynthetic pathway (from acetyl‐CoA to farnesyl pyrophosphate then to DON) have been comprehensively investigated (Figure 1) (Alexander et al., 2011; Gardiner et al., 2009). Previous studies have demonstrated that genes involved in trichothecene biosynthesis reside at different chromosomal loci. Nearly all *Tri* genes are derived from the core 25‐kb region of the *Tri5*‐cluster that includes *Tri4*, *Tri5*, *Tri6*, and *Tri10* (Alexander et al., 2011; Kimura et al., 2003). *Tri1* and *Tri101* genes reportedly have been found at separate loci (Alexander et al., 2011; Brown et al., 2003; Peplow et al., 2003).

Notably, recent studies of the enzymes involved in the DON metabolic pathway in vivo revealed that enzymes Tri1, Tri4, and Hmr1 colocalize in the remodelled endoplasmic reticulum (ER). Based on these studies, a novel and unique subcellular organization containing DON biosynthetic enzymes, the “toxisome”, was proposed in *Fusarium* species (Boenisch et al., 2017; Menke et al., 2013). The myosin I‐actin cytoskeleton, which has been identified as a toxisome component, was also determined to play an indispensable role in toxisome formation in *Fusarium* species (Tang et al., 2018).

Accumulated evidence suggests that the microtubule (MT) cytoskeleton plays a vital role in the sequential organization of metabolic enzymes (An et al., 2010; Volker et al., 1995). A previous study showed that the purine biosynthetic metabolons, the purinosomes, were embedded with the MT networks in HeLa cells and that the MT cytoskeleton guided the spatial distribution of purinosomes through the embedded locate mode (An et al., 2010). In our current study, we questioned whether *F. graminearum* toxisome is associated with the MT cytoskeleton and how this association regulates mycotoxin biosynthesis. To test this hypothesis, we fused calonectrin oxygenase (Tri1) with green fluorescent protein (GFP) as a toxisome marker and investigated the structural and functional relationships between the toxisome and the MT cytoskeleton.

*Saccharomyces cerevisiae* and most other phytopathogens have three types of tubulins (α_1_, α_2_, and β) (Duan et al., 2015; Liu et al., 2013; Neff et al., 1983; Schatz et al., 1986), whereas *F. graminearum* contains four types of tubulins: α_1_, α_2_, β_1_, and β_2_ (Hu et al., 2015; Zhou et al., 2016). To visualize and manipulate the MT cytoskeleton in the presence of toxisomes, we used *F. graminearum* tubulin β_1_ chain (β_1_ tubulin) fused with red fluorescent protein (RFP) as an MT marker and the hyphae were treated with a high concentration of carbendazim, the most widely used fungicide in the field, which directly binds to MT to disrupt spindle formation. In addition, we studied the functional connection between the toxisome and the MT by generating a knockout mutation of each *F. graminearum* tubulin subunit (α_1_, α_2_, β_1_, and β_2_). We also measured DON production to illuminate the metabolic functionality of toxisomes in the presence or absence of carbendazim and each tubulin subunit. Based on our findings, we propose that the α_1_–β_2_ tubulin heterodimer is the scaffold that helps spatial distribution and functionality of active toxisomes in *F. graminearum*.

## 2 RESULTS

### 2.1 Two enzymes in mevalonate metabolism are localized in the *F. graminearum* toxisome

**FIGURE 2:**
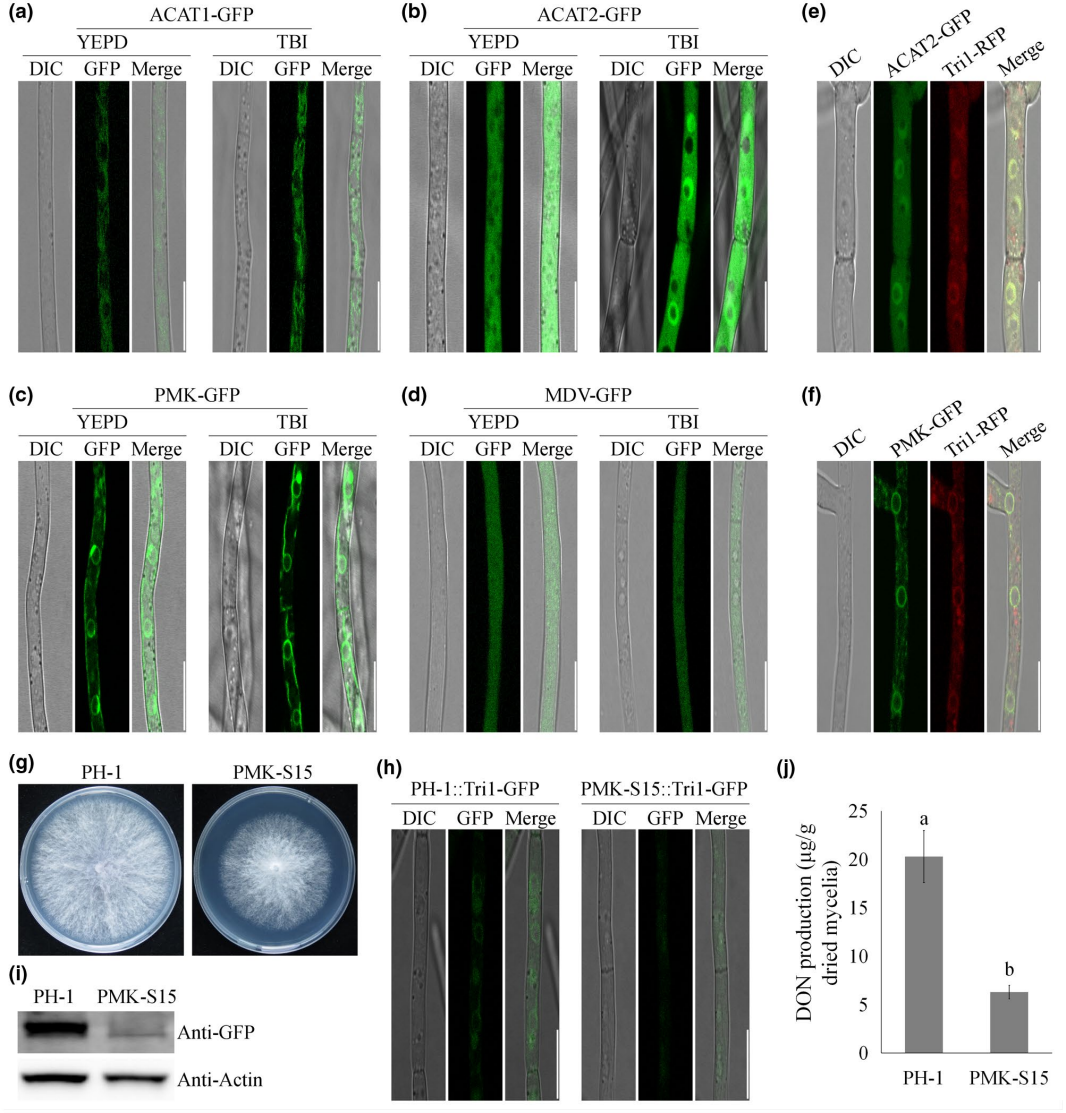
Subcellular localization of four enzymes involved in mevalonate metabolism. (a)–(d) Localization of enzymes in hyphae of PH‐1 tagged with green fluorescent protein (GFP) growing in toxin‐noninducing yeast extract peptone dextrose (YEPD) medium and trichothecene biosynthesis induction (TBI) medium for 2 days. Bar = 10 μm. (e) and (f) FgACAT2‐GFP and FgPMK‐GFP were colocalized with Tri1‐RFP under toxin‐inducing conditions at 28 °C for 2 days. Bar = 10 μm. (g) FgPMK‐S15 exhibited reduced hyphal growth rate on potato dextrose agar. (h) Toxisome assembly was reduced in FgPMK‐S15 grown in TBI medium. Bar = 10 μm. (i) The accumulation of Tri1‐GFP protein was dramatically reduced in FgPMK‐S15. (j) Thedeoxynivalenol (DON)production of FgPMK‐S15 significantly decreased in comparison with PH‐1. Values on the bars followed by the same letter are not significantly different at α =.05 according to Fisher's LSD test

While enzymes involved in DON biosynthesis have been studied intensively, the subcellular metabolon for de novo DON biosynthesis, the toxisome in *F. graminearum*, was only proposed recently (Menke et al., 2013). Other components were also found localized in the toxisome, including a mevalonate biosynthetic enzyme Hmcr, two trichothecene biosynthetic pathway enzymes Tri1 and Tri4, ribosomal protein Asc1, and cytoskeleton proteins myosin I and actin (Boenisch et al., 2017; Tang et al., 2018). To further characterize the toxisome components, we selected four mevalonate biosynthetic enzymes and tagged them with GFP in the wild‐type strain PH‐1: two acetyl‐CoA acetyltransferases, ACAT1 (FGSG_05087) and ACAT2 (FGSG_09321), phosphomevalonate kinase PMK (FGSG_09764), and diphosphomevalonate decarboxylase MDV (FGSG_10424). The strains expressing ACAT1‐GFP, ACAT2‐GFP, and MDV‐GFP showed cytoplasmic fluorescence only during growth in toxin‐noninducing yeast extract peptone dextrose (YEPD) medium (Figure 2a,b,d). In toxin‐inducing conditions, ACAT2‐GFP was partially localized to yet‐to‐be‐determined spherical structures (Figure 2b). We stained the ACAT2‐GFP strain with the vacuole‐labelling dye 7‐amino‐4‐chloromethylcoumarin and verified that ACAT2 does not localize to the vacuoles (Figure S1). Interestingly, stronger fluorescence from PMK‐GFP was localized to the spherical structures under toxin‐noninducing and toxin‐inducing conditions (Figure 2c). Furthermore, ER‐tracker blue and 4’,6‐diamidino‐2‐phenylindole staining indicated that PMK‐GFP is not only associated with the ER but also with the perimeter of the nucleus (Figure S2). To determine whether these two enzymes (ACAT2 and PMK) localize to the toxisomes in toxin‐inducing conditions, we tagged Tri1 with an RFP in ACAT2‐GFP and PMK‐GFP strains. Tri1‐RFP colocalized with ACAT2‐GFP and PMK‐GFP in trichothecene biosynthesis induction (TBI) medium to some degree, as shown in Figure 2e,f.

To verify the role of these mevalonate biosynthetic enzymes in the toxisome assembly, we performed *FgPMK* knockdown by transforming the recombinant plasmid pSilent‐FgPMK, conferring the hairpin RNA of an *FgPMK* fragment (485 bp), into the wild‐type strain PH‐1. Among the 12 tested transformants, eight showed decreased expression of *FgPMK* in comparison with the wild‐type progenitor (Figure S3). Among these transformants, FgPMK‐S15 showed the lowest *FgPMK* expression (15% of the wild‐type strain PH‐1), and was thus selected for further experiments. The mutant exhibited slightly reduced growth on potato dextrose agar (PDA) (Figure 2g) and, as expected, FgPMK‐S15 mycelia formed only faint toxisomes in toxin‐inducing conditions (Figure 2h). The translation level of Tri1‐GFP protein in FgPMK‐S15 was assessed by western blot analysis, and the result was consistent with fluorescent signals (Figure 2i). The mutant FgPMK‐S15 also exhibited significantly reduced DON production (Figure 2j). Taken together, these results suggest that mevalonate biosynthetic enzymes ACAT2 and PMK localize in toxisome under toxin‐inducing conditions and play an important role in DON biosynthesis.

### 2.2 Microtubule‐associated toxisome assembly

**FIGURE 3:**
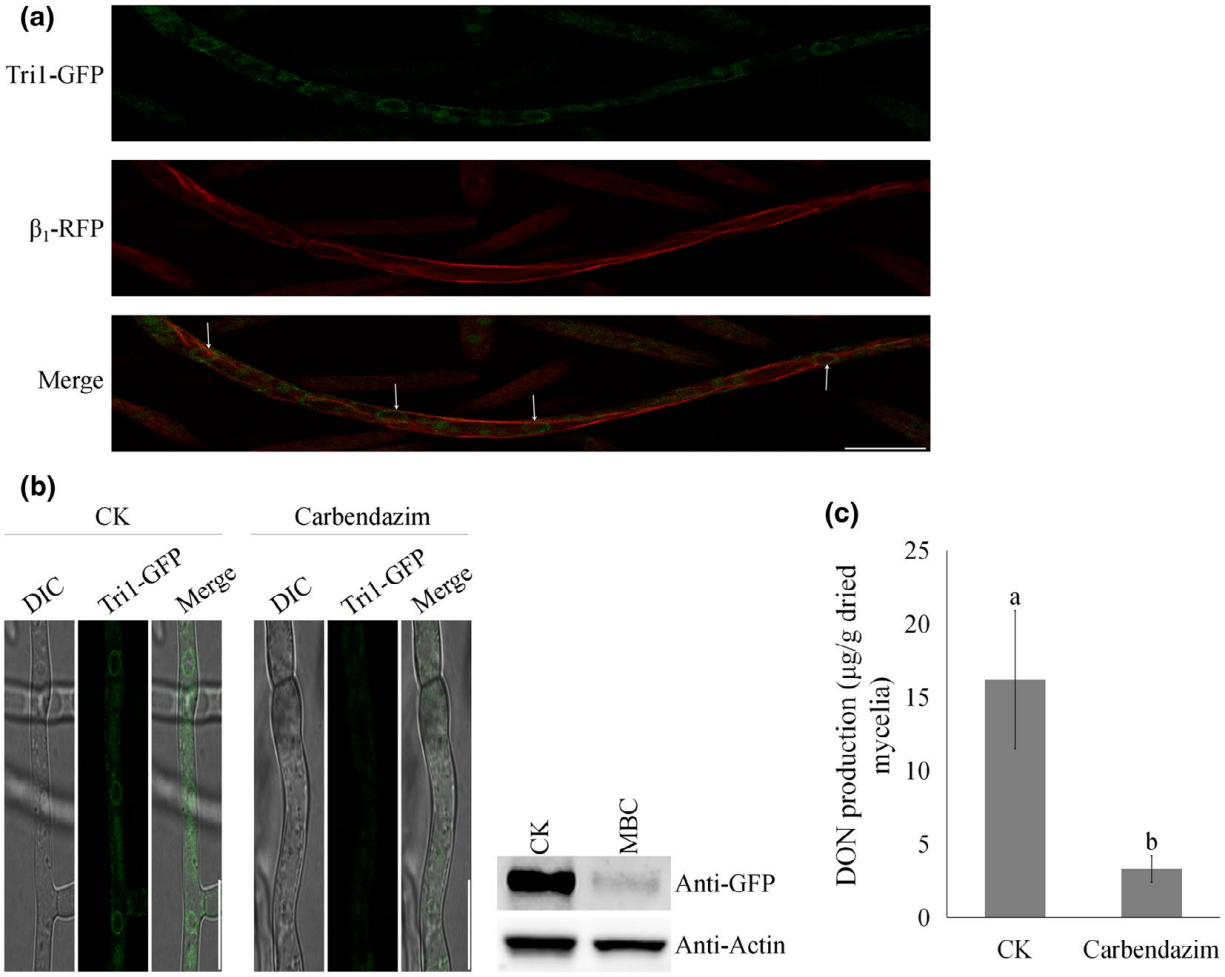
Microtubules regulate toxisome assembly under toxin‐inducing conditions. (a) Subcellular localization of toxisomes associated with microtubule filaments in hyphae grown in trichothecene biosynthesis induction (TBI)medium. Bar = 10 μm. (b) Effects of carbendazim on toxisome assembly. After growth in TBI medium for 24 hr, 1.2 μg/ml carbendazim was added and incubated for another 24 hr. Bar = 10 μm. (c) Effects of carbendazim (1.2 μg/ml) ondeoxynivalenol (DON)production of hyphae grown in TBI medium. Values on the bars followed by the same letter are not significantly different at α = .05 according to Fisher's LSD test

To investigate whether the MT cytoskeleton is involved in the toxisome assembly, we first constructed a strain bearing β_1_‐RFP and Tri1‐GFP in the wild‐type progenitor. As indicated in Figure 3a, toxisomes were found to be associated with MT filaments in *F. graminearum* hyphae. Furthermore, we examined how toxisome assembly responds to the small‐molecule fungicide carbendazim, which targets the MT cytoskeleton and interferes with its formation during mitosis. After treatment for 24 hr with 1.2 μg/ml carbendazim (approximately EC_95_ against mycelial growth), Tri1‐GFP only displayed weak signals when compared with the control samples (Figure 3b, left panel; Figure S4). The expression of Tri1‐GFP was verified by the western blot assay, and the intensity of the GFP band was dramatically reduced in the samples treated with carbendazim (Figure 3b, right panel). Accordingly, there was a significant reduction in DON production after treatment with carbendazim (Figure 3c). Collectively, the assembly of toxisome is associated with MT, and the disruption of MT by carbendazim impaired the cluster assembly of toxisome and DON biosynthesis in toxin‐inducing conditions.

### 2.3 ɑ_1_ tubulin participates in toxisome assembly

**FIGURE 4:**
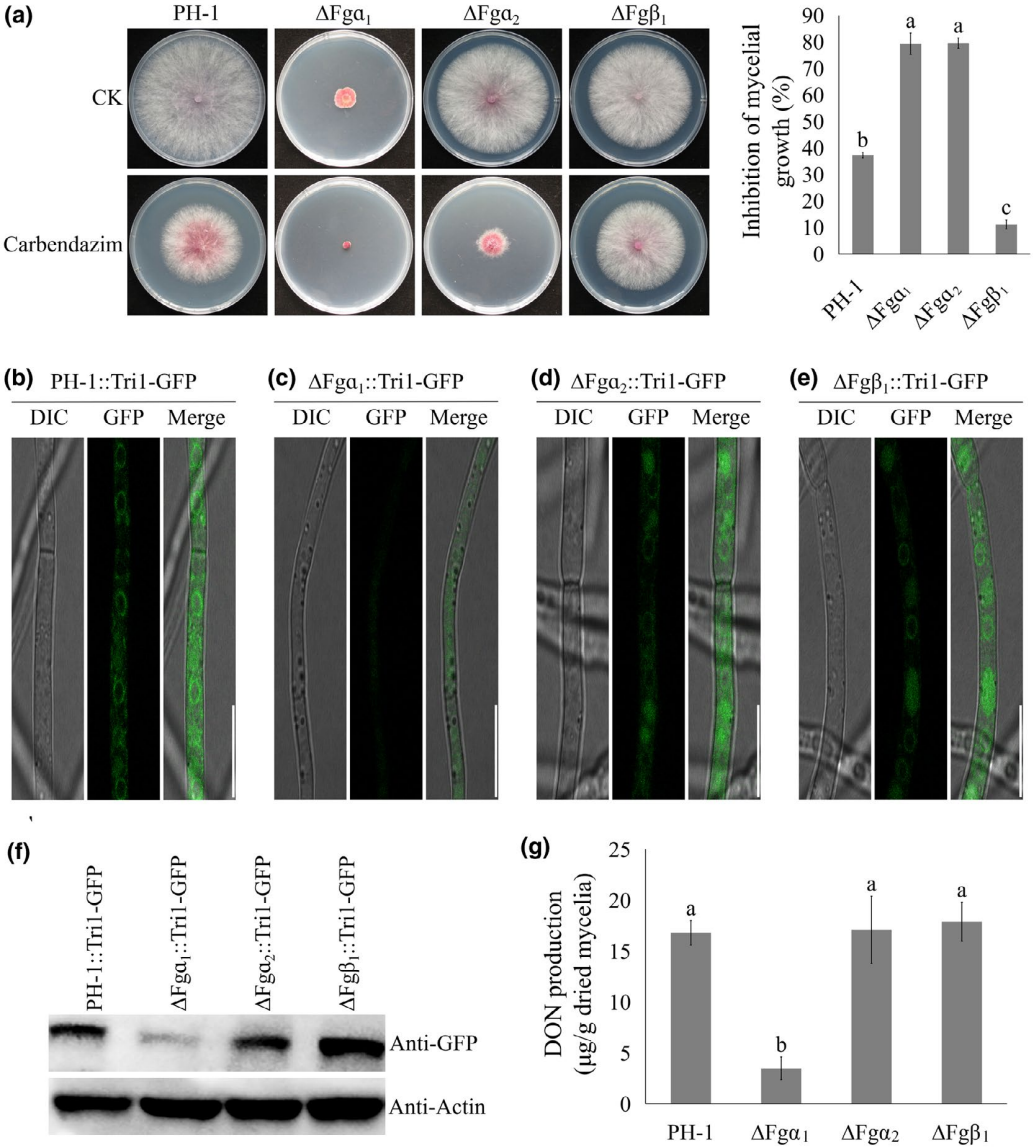
α_1_tubulin is involved in toxisome assembly. (a) The sensitivityof ΔFgα_1_, ΔFgα_2_, and ΔFgβ_1_towards carbendazim. All strains were cultured on potato dextrose agar supplemented with 0.5 μg/ml carbendazim (left panel). Mycelial growth inhibition of all strains by carbendazim was quantified (right panel). (b)–(e) Toxisome assembly in PH‐1, ΔFgα_1_, ΔFgα_2_, and ΔFgβ_1_. The images were taken after all strains labelled withTri1‐GFP were incubated in trichothecene biosynthesis induction (TBI) medium for 2 days. Bar = 10 μm. (f) The translation level of Tri1‐GFP protein in PH‐1, ΔFgα_1_, ΔFgα_2_, and ΔFgβ_1_. (g)Deoxynivalenol (DON)production in PH‐1, ΔFgα_1_, ΔFgα_2_, and ΔFgβ_1_. Values on the bars followed by the same letter are not significantly different at α = .05 according to Fisher's LSD test

To better define the impact of the MT cytoskeleton in toxisome assembly, we investigated the role of each MT component. First, we deleted ɑ_1_ tubulin in the wild‐type strain PH‐1, and Tri1‐GFP plasmid was subsequently introduced into the deletion mutant ∆Fgα_1_. All transformants were screened under a confocal microscope and further verified by western blot assay (data not shown). The results showed that ∆Fgα_1_ showed drastically impaired hyphal growth when compared with PH‐1. When treated with 0.5 μg/ml carbendazim, ∆Fgα_1_ exhibited increased drug sensitivity against the fungicide (Figure 4a). As shown in Figure 4b,c, the Tri1‐GFP signals sharply decreased in ∆Fgα_1_. Furthermore, western blot assays showed that the expression of Tri1‐GFP protein was significantly lower in ∆Fgα_1_ compared with PH‐1 in TBI medium (Figure 4f). Furthermore, when we measured DON production in equal mycelial mass, significantly less mycotoxin was produced in ∆Fgα_1_ in comparison with PH‐1 (Figure 4g). These data on the α_1_ tubulin deletion mutant are in line with the wild‐type phenotypes when treated with carbendazim described earlier, and indicate that α_1_ tubulin plays a vital role in organizing the toxisome assembly in *F. graminearum*.

### 2.4 α_2_ and β_1_ tubulin are dispensable for DON biosynthesis

To test whether or not α_2_ tubulin is necessary for toxisome assembly, we deleted α_2_ tubulin in the wild‐type strain PH‐1. The results revealed that ∆Fgα_2_ showed hyphal growth similar to PH‐1 but exhibited increased sensitivity to the MT inhibitor carbendazim (Figure 4a). When the toxisome assembly in ∆Fgα_2_::Tri1‐GFP mutant was examined, the Tri1‐GFP signals showed no difference in ∆Fgα_2_ compared with the wild type (Figure 4b,d). Western blot assays also confirmed that the expression of the Tri1‐GFP protein in ∆Fgα_2_ was similar to the wild‐type progenitor (Figure 4f). Furthermore, DON production in ∆Fgα_2_ was similar to the wild type (Figure 4g). We concluded that α_2_ tubulin is not critical for toxisome assembly in *F. graminearum*.

We also constructed a deletion mutant of β_1_ tubulin to investigate toxisome association. The mutant ∆Fgβ_1_ showed similar hyphal growth compared with PH‐1 and decreased sensitivity to the MT inhibitor carbendazim (Figure 4a). In addition, the Tri1‐GFP signals and the expression of Tri1‐GFP protein showed no difference between ∆Fgβ_1_ and the wild‐type progenitor (Figure 4b,e,f). Furthermore, DON production was similar in ∆Fgβ_1_ and the wild type (Figure 4g). We concluded that β_1_ tubulin is not involved in DON biosynthesis in *F. graminearum*.

### 2.5 β_2_ tubulin‐associated toxisome assembly

**FIGURE 5:**
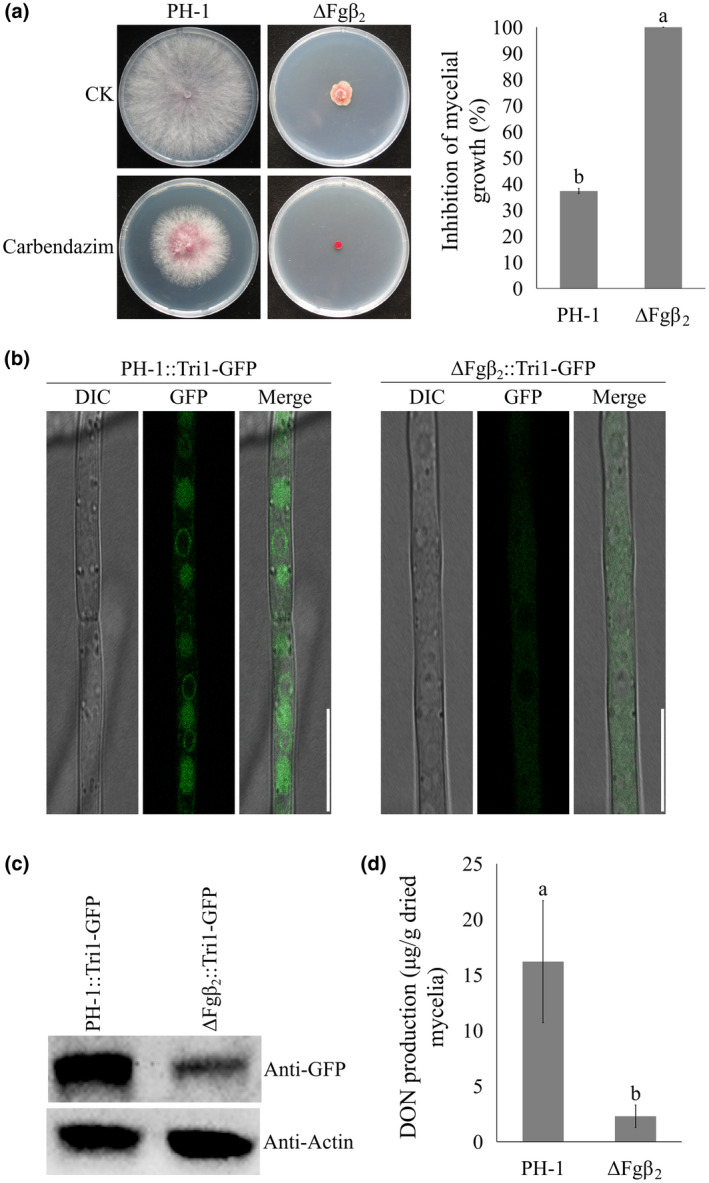
Deletion of β_2_tubulin impairs toxisome assembly. (a) The sensitivity of PH‐1 and ΔFgβ_2_ towards carbendazim.All strains were cultured on potato dextrose agar supplemented with 0.5 μg/ml carbendazim (left panel). Mycelial growth inhibition of all strains by carbendazim was quantified (right panel). (b) Toxisome assembly was not detected in ΔFgβ_2_. Bar = 10 μm. (c) The translation level of Tri1‐GFP proteins was determined by western blot assay. (d) Production of deoxynivalenol (DON) in PH‐1 and ΔFgβ_2_ after being cultured in trichothecene biosynthesis induction medium for 7 days. Values on the bars followed by the same letter are not significantly different at α = .05 according to Fisher's LSD test

Previous studies showed that the carbendazim‐resistant mutations in β_2_ tubulin significantly increased DON production in *F. graminearum* (Zhang et al., 2009; Zhou et al., 2020), suggesting that β_2_ tubulin is involved in the regulation of DON biosynthesis. In this study, we further investigated whether β_2_ tubulin, one of the key MT components, is involved in toxisome assembly. As anticipated, the β_2_ tubulin deletion mutant ∆Fgβ_2_ showed significantly reduced hyphal growth and increased drug sensitivity to the tubulin inhibitor carbendazim similar to that observed in ∆Fgα_1_ (Figure 5a). Using fluorescent live cell imaging, we found that toxisome assembly was severely impaired in ∆Fgβ_2_ with weak fluorescent signals loosely dispersed in hyphae (Figure 5b). In addition, western blot analysis of Tri1‐GFP protein in the wild‐type PH‐1 and ∆Fgβ_2_ was in agreement with the fluorescent signals (Figure 5c). Furthermore, DON production in the mutant was drastically lower than wild type in toxin‐inducing conditions (Figure 5d). These results show that β_2_ tubulin is indispensable for toxisome assembly.

### 2.6 α_1_–β_2_ tubulin dimer facilitates toxisome assembly by providing a scaffold

**FIGURE 6:**
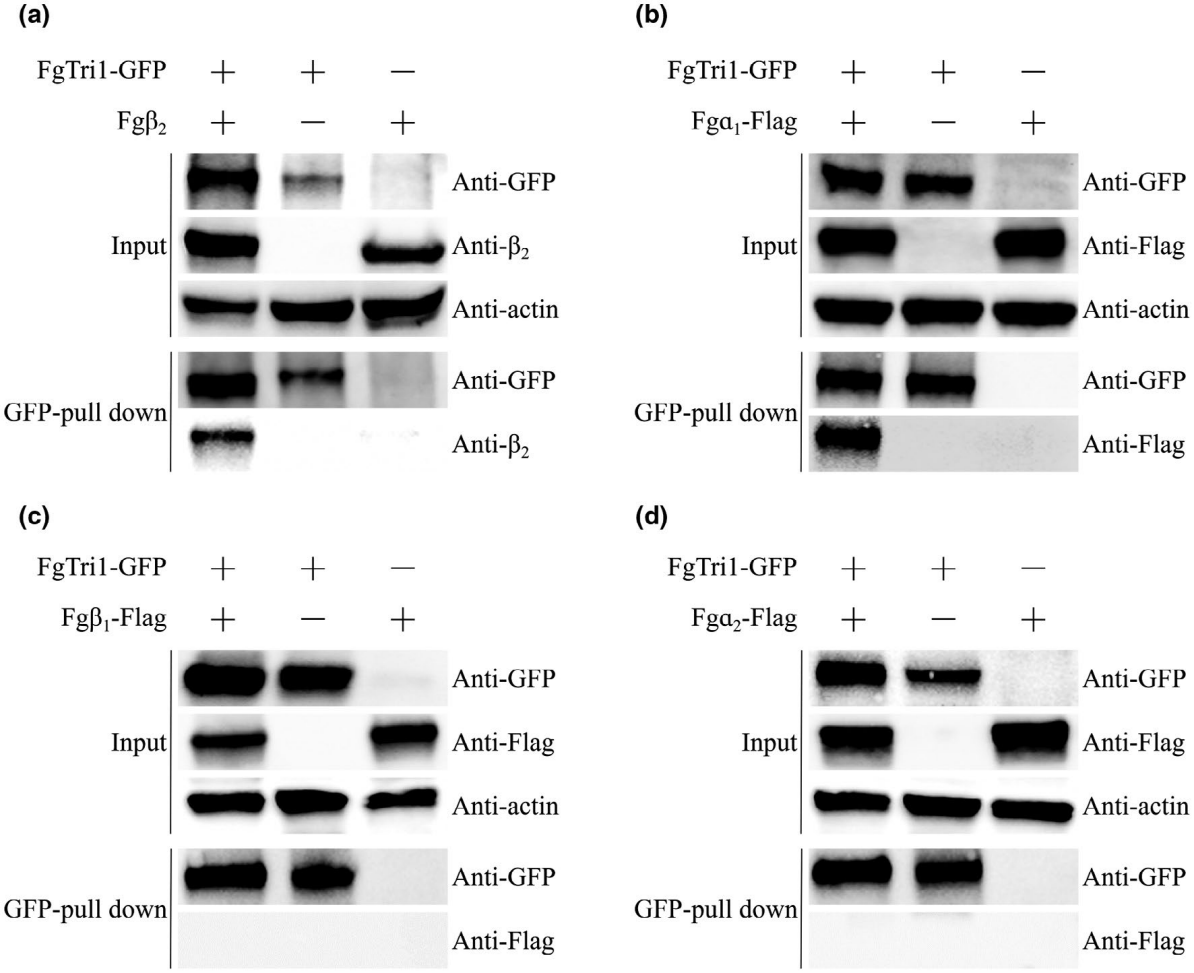
Coimmunoprecipitation analysis of the interaction between FgTri1 and tubulin Fgβ_2_(a), Fgα_1_(b), Fgβ_1_(c), or Fgα_2_(d). Total proteins extracted from the strain bearing Tri1‐GFP and tubulin‐FLAG/tubulin constructs or a single construct (Tri1‐GFP or tubulin‐FLAG/tubulin) were subjected to sodium dodecyl sulphate‐polyacrylamide electrophoresis, and immunoblots were incubated with anti‐GFP, anti‐FLAG/anti‐β_2_,and anti‐actin antibodies (input panel). All protein samples were pulled down using anti‐GFP magnetic beads and then detected with anti‐GFP and anti‐FLAG/anti‐β_2_antibodies (GFP pull‐down panel)

In the current study, ∆Fgα_1_ and ∆Fgβ_2_ mutants showed consistent defects in hyphal growth, drug sensitivity, toxisome assembly, and DON biosynthesis. Therefore, we hypothesized that (a) α_1_ tubulin is associated with β_2_ tubulin and (b) α_1_ and β_2_ tubulin are associated with toxisome components in *F. graminearum*. To test our hypotheses, we first constructed a strain bearing α_1_‐GFP and β_2_‐RFP. Using fluorescent live cell imaging, we showed that Fgα_1_‐GFP colocalizes with Fgβ_2_‐RFP (Figure S5). Subsequently, we constructed a strain that was dual‐labelled with either Fgɑ_1_‐3 × FLAG and Tri1‐GFP or Fgβ_2_ and Tri1‐GFP, followed by culturing in TBI medium for 48 hr. The mycelial samples then were harvested for protein extraction and coimmunoprecipitation (Co‐IP) assays. The results verified the interactions between Tri1 and β_2_ tubulin as well as Tri1 and α_1_ tubulin (Figure 6a,b). Furthermore, we demonstrated that Tri1 does not interact with α_2_ or β_1_ tubulin (Figure 6c,d), which is consistent with their roles in toxisome assembly and DON biosynthesis. These results provide a strong argument for the critical role α_1_–β_2_ tubulin heterodimer plays in toxisome assembly, and we postulate that it serves as the scaffold for the spatial organization and structure stabilization of toxisomes in *Fusarium* species.

## 3 DISCUSSION

The formation of metabolic multienzyme complexes, metabolons, is thought to promote metabolic processes and provide a coordinated system for the cell to effectively control metabolism (Kohnhorst et al., 2017; Thomas et al., 2017). Recently, the existence of metabolons in mammalian cells has come to light with the development of advanced cell‐based techniques. Accumulating evidence has indicated that various metabolons exist in cell metabolisms, such as glycosome in glycolysis, pyruvate dehydrogenase complex (PDC) in the tricarboxylic acid (TCA) cycle, and purinosome in the purine biosynthesis pathway (An et al., 2010; Haanstra et al., 2016; Haggie & Verkman, 2002). Additionally, systems biology combined with omics strategies has shown that metabolons often interact with other subcellular components, particularly MTs, to maximize their functions (French et al., 2016; Ori et al., 2016; Rhee et al., 2013). In HeLa cells, purinosomes were embedded in the MT network and this spatial distribution significantly accelerated enzyme activity (An et al., 2010). In *Fusarium* species, a novel secondary metabolism complex containing several DON biosynthesis enzymes, the toxisome, was found recently (Boenisch et al., 2017; Menke et al., 2013). Moreover, myosin I‐actin cytoskeleton was shown to play a vital role in toxisome formation (Tang et al., 2018). Because the biochemical and physiological connections between toxisome and the MT cytoskeleton have not been investigated, our key motivation for this study was to better define their functional correlation.

In eukaryotic cells, the MT cytoskeleton is associated with various cellular processes, including cell motility, mitosis, polarity, and vesicle traffic. By conducting a fluorescent live cell imaging of the MT cytoskeleton in the presence of toxisomes, we provide clear evidence that toxisomes are associated with the network of MTs (Figure 2a). Our studies found that DON production was significantly increased in β_2_ tubulin carbendazim‐resistant mutants (Zhou et al., 2020), indicating that β_2_ tubulin is involved in DON biosynthesis. In addition, Tang and colleagues showed that carbendazim treatment (approximately EC_50_ against mycelial growth) increased DON biosynthesis in *F. graminearum* (Tang et al., 2018). We obtained similar results when we treated our wild‐type strain with carbendazim in this study (Figure S6). However, we found that disruption of the MT network by the addition of a high concentration of carbendazim (approximately EC_95_ against mycelial growth) was sufficient to dissociate toxisomes in *F. graminearum* (Figure 2b). Furthermore, the architecture and function of MTs are regulated by microtubule‐associated proteins (MAPs). Among various MAPs, the end‐binding protein 1 (EB1) has been well studied in mammalian cells, yeast, and phytopathogens including *F. graminearum*. In yeasts, the homologs of EB1 have been proven to regulate microtubule dynamics, chromosome stability, and cell polarization (Schwartz et al., 1997). In *F. graminearum*, the MT network exhibited unstable organization in the absence of EB1 (Liu et al., 2017). Similarly, the homologs of EB1 are involved in regulating MT integrity and cell formation in *Schizosaccharomyces pombe* (Beinhauer et al., 1997). As expected, toxisome assembly and DON biosynthesis were severely impaired in the EB1 deletion mutant (Liu et al., 2017). All these results indicate that the integrity of the MT network is important for toxisome assembly and DON production in *Fusarium* species. Therefore, we hypothesized that the spatial distribution of toxisomes is closely connected with MT networks. Our experiments using carbendazim treatment and MT mutants clearly demonstrated that MTs serve critical roles in DON biosynthetic enzyme organization under toxin‐inducing conditions. When the integrity of the MT cytoskeleton was impaired, the proper proximity of DON biosynthetic enzymes to one another would be disrupted, which would lead to a significant decrease in DON metabolic efficiency. Thus, this cell‐based toxisome activity experiment indeed supports our hypothesis that MTs are functionally necessary for toxisome assembly in *F. graminearum*.

Previous studies in *S. cerevisiae* showed that β‐tubulin is directly associated with α tubulin (α_1_ and α_2_) to form a tubulin heterodimer, which polymerizes to form MTs (Mandelkow & Mandelkow, 1989; McKean et al., 2001). Four tubulin subunits (α_1_, α_2_, β_1_, and β_2_) are found in *F. graminearum*, and previous studies showed that α_1_ and β_2_ tubulin are involved in DON biosynthesis (Hu et al., 2015; Wang et al., 2019). In addition, Fgα_1_ was captured by Fgβ_2_ in our affinity capture–mass spectrometry (ACMS) assays (authors’ unpublished data). In this study, we further investigated and confirmed the association of the toxisome with each tubulin subunit. The dissociation of the toxisome was evident in the absence of α_1_ or β_2_ tubulin whereas deletion of α_2_ or β_1_ tubulin did not affect toxisome assembly. In addition, Tri1‐GFP translation and DON production were also significantly reduced in the absence of α_1_ or β_2_ tubulin, which is consistent with toxisome assembly. Furthermore, we demonstrated that α_1_ tubulin is directly connected with β_2_ tubulin in *F. graminearum*. These results indicate that ɑ_1_ tubulin associates with β_2_ tubulin to form the α_1_–β_2_ tubulin heterodimer, thereby participating in toxisome assembly and DON biosynthesis in *F. graminearum*.

Numerous proteins have been reported to bind with MTs or their subunits to some degree, which can alter their activity in comparison with non‐MT bound proteins. These proteins include glycolytic enzymes, purine biosynthesis enzymes, mitochondrial outer membrane protein voltage‐dependent anion channel (VDAC), and hypoxia‐inducible factor (HIF)‐1. For example, hexokinase (HK) and pyruvate kinase (PK) have been demonstrated to colocalize with MTs in animal cells, which resulted in greater enzyme activity (Walsh et al., 1989; Wagner et al., 2001). In HeLa cells, purine biosynthetic enzymes embedded in the MT network in purine‐depleted conditions, which accelerated the rate of de novo purine synthesis (An et al., 2010). Results showed that the transport of ions and metabolites across the outer membrane of the mitochondria is controlled by the VDAC, and tubulin dimers can regulate the switching of VDAC (Lemasters & Holmuhamedov, 2006; Shoshan‐Barmatz et al., 2010). Additionally, a glycolytic metabolon is known to be stabilized by the actin cytoskeleton in *S. cerevisiae* (Araiza‐Olivera et al., 2013). In the present study, we showed that both α_1_ and β_2_ tubulin interact with Tri1, an important component of the toxisome. We postulate that this interaction stabilizes the structure of toxisome, thereby facilitating DON biosynthesis. When the structure of α_1_–β_2_ tubulin heterodimer was impaired, the toxisome could not form in *F. graminearum*, which is deleterious to maintaining the proximity and high activity of all DON biosynthetic enzymes.

The ER is a complex organelle that is involved in lipid and protein biosynthesis and calcium regulation as well as interactions with other organelles. The structure and distribution of ER is regulated by various membrane proteins and interactions with cytoskeleton and other organelles. Recent reports have shown that toxisome enzymes are colocalized at the reorganized ER under toxin‐inducing conditions in *F. graminearum* (Boenisch et al., 2017). However, the molecular mechanism for ER reorganization remains to be elucidated. In HeLa cells, the tubule‐to‐sheet transition in ER was regulated by MTs, and nocodazole (an MT depolymerization drug) treatment could disrupt this transition (Lu & Kirchhausen, 2012). Additionally, ER tubules can be formed by membranes sliding along MTs or attached to polymerizing MTs (Waterman‐Storer & Salmon, 1998). Based on this evidence, we speculated that MTs might be involved in ER structure formation in *F. graminearum*. The interactions between MT and ER under toxin‐inducing conditions remains unknown, however, and need further research.

**FIGURE 7:**
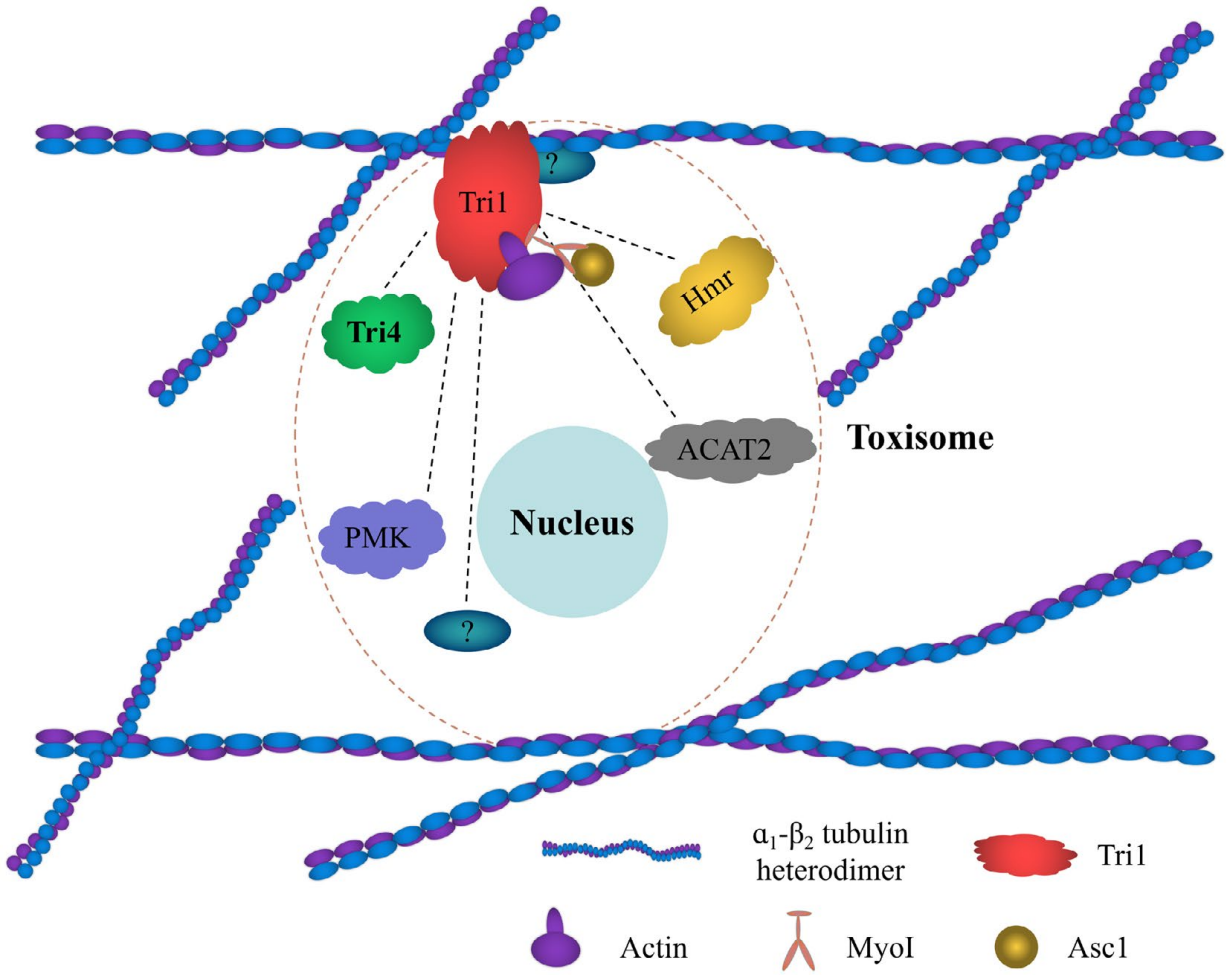
A proposed model presenting the role of the α_1_–β_2_tubulin heterodimer in toxisome assembly. In toxin‐inducing conditions, the α_1_–β_2_tubulin heterodimer interacts with the trichothecene biosynthetic enzyme Tri1, providing a scaffold for Tri1 attachment and organizing enzymes in the toxisome sequentially, and subsequently facilitating toxisome assembly and DON biosynthesis. High concentration carbendazim and microtubule‐associated proteins can disrupt toxisome assembly by impairing the integrity of the α_1_–β_2_tubulin heterodimer, thereby decreasing DON biosynthesis in*F. graminearum*

In summary, our study supports a model of MT heterodimer interacting with Tri1, in a “piggy‐backing” way, and α_1_–β_2_ tubulin and Tri1 serve as scaffold and anchor, respectively, to further organize toxisome assembly and stabilize toxisome structure, which ultimately activates DON biosynthesis in *F. graminearum* (Figure 7). However, the complex protein–protein interaction network among various toxisome components and their association with α_1_–β_2_ tubulin heterodimer remains inconclusive. Thus, it would be worthwhile studying further the protein–protein interaction network of the toxisome and its interactions with MTs to improve our insight into DON regulation mechanism in *Fusarium* species.

## 4 EXPERIMENTAL PROCEDURES

### 4.1 Strains and culture assays

*F. graminearum* wild‐type strain PH‐1 was used for the construction of the derived mutants in this study. All strains used in this study were grown and evaluated at 25 °C on PDA for mycelial growth and fungicide sensitivity assays. Mung bean broth medium was used for conidia culture (Zhang et al., 2016). For DON production analysis or toxisome observation, all strains were grown in TBI medium at 28 °C with agitation (175 rpm) in the dark for 7 days or 2 days, respectively (Tang et al., 2018). All experiments were repeated three times independently.

### 4.2 Strain construction

The strains ΔFgα_1_, ΔFgα_2_, and ΔFgβ_1_ were constructed using the protocol described previously (Liu et al., 2013; Qiu et al., 2012). All targeted open reading frames (ORFs) were replaced with the *HPH‐HSV‐tk* fragment, and all transformants were analysed by PCR assays with corresponding primers and by quantitative PCR assays. Fgα_1_‐3 × FLAG and Fgβ_1_‐RFP fusion cassette were constructed following the protocol described previously (Zhang et al., 2016). Tri1‐GFP fusion cassette was constructed as described previously and then transformed into the corresponding mutants (Bruno et al., 2004).

### 4.3 Analysis of DON production

To measure DON production, PH‐1 and all mutants were grown in liquid TBI medium. After incubation at 28 °C for 7 days in the dark, the mycelia and 1 ml of TBI medium were harvested. Thereafter, DON was assayed with a competitive enzyme‐linked immunosorbent assay detection plate kit (Wise) according to the protocol described in previous studies (Duan et al., 2018; Li et al., 2019).

### 4.4 Microscopic examinations

The localization of Tri‐GFP protein was observed with a TCS SP5 confocal microscope (Leica). For examination of toxisome assembly patterns in PH‐1 and derived mutants, all strains labelled with Tri1‐GFP were cultured in TBI medium for 2 days before observation. The following parameters for confocal microscopy were used: Plan‐Neofluar 100×/1.30 oil DIC objective, laser at 488 nm at 30% power for green fluorescence or at 561 nm at 40% power for red fluorescence, pinhole 100 μm, and digital gain 1.00.

### 4.5 Western blotting analysis

All transformants were cultured in liquid TBI medium for 2 days, thereafter the mycelia were harvested for protein extraction. The protein extraction and western blotting analysis were performed as previously described (Gu et al., 2015; Zhou et al., 2020). Ten microlitres of protein sample was analysed by western blotting. The monoclonal anti‐GFP antibody 300943 (Zenbio) was used at a 1:1,000 dilution ratio to detect Tri‐GFP fusion protein. All samples were also assayed with monoclonal anti‐actin antibody 700068 (Zenbio) as a reference. The intensity of immunoblot bands was quantified using Gel‐Pro analyser software. All experiments were repeated three times.

### 4.6 Co‐IP assays

The Tri1‐GFP or α_1_‐3 × FLAG‐fusion constructs were verified by DNA sequencing and transformed into PH‐1 or corresponding mutants. Transformants expressing Tri1‐GFP and β_2_ tubulin or Tri1‐GFP and α_1_‐3 × FLAG were confirmed by western blotting analysis. In addition, the transformants expressing a single tag protein were used as references. For Co‐IP assays, magnetic beads (Bio‐Rad) were first incubated with the monoclonal anti‐GFP antibody 300943 following the manufacturer's protocol. Thereafter, the magnetic beads were incubated with total protein samples. Protein samples (10 μl) eluted from magnetic beads were analysed by western blotting with a polyclonal anti‐β_2_ antibody IF11 (Zhou et al., 2016) or a polyclonal anti‐FLAG A9044 (Zenbio). Total protein samples were further assayed with monoclonal anti‐actin antibody 700068 as a reference. All experiments were repeated twice.

## Supporting information


**FIGURE S1** FgACAT2 is not localized to the vacuole. Mycelia of FgACAT2 were grown in YEPD and stained with 7‐amino‐4‐chloromethylcoumarin. Bar = 10 μm

**FIGURE S2** FgPMK is associated with the ER. Mycelia of FgPMK were grown in YEPD and stained with 4’,6‐diamidino‐2‐phenylindole (a) and ER‐Tracker (b). Bar = 10 μm

**FIGURE S3** Transcription level of *FgPMK* in different *FgPMK* silencing transformants

**FIGURE S4** Time course analysis of expression of Tri1‐GFP in toxin‐inducing conditions after carbendazim treatment

**FIGURE S5** Fgα_1_‐GFP was colocalized with Fgβ_2_‐RFP in the hyphae of *Fusarium graminearum*. Bar = 10 μm

**FIGURE S6** The effect of carbendazim treatment (0.5 μg/ml) on DON accumulation. (a) Effects of carbendazim on toxisome assembly. After growth in TBI medium for 24 hr, 0.5 μg/ml carbendazim was added and incubated for another 24 hr. Bar = 10 μm. (b) Effects of carbendazim (0.5 μg/ml) on DON production of hyphae grown in TBI medium. Values on the bars followed by the same letter are not significantly different at α = 0.05 according to Fisher’s LSD test

**TABLE S1** Wild‐type and mutants of *Fusarium graminearum* used in this study

**TABLE S2** Primers used in this study

## Data Availability

The data that support the findings of this study are available from the corresponding author upon reasonable request.
