# Supplementary figures and images for: Microtubule‐assisted mechanism for toxisome assembly in Fusarium graminearum
Source: Mol Plant Pathol. 2020 Nov 17;22(2):163–74. doi: 10.1111/mpp.13015 (PMC7814972; doi:10.1111/mpp.13015)

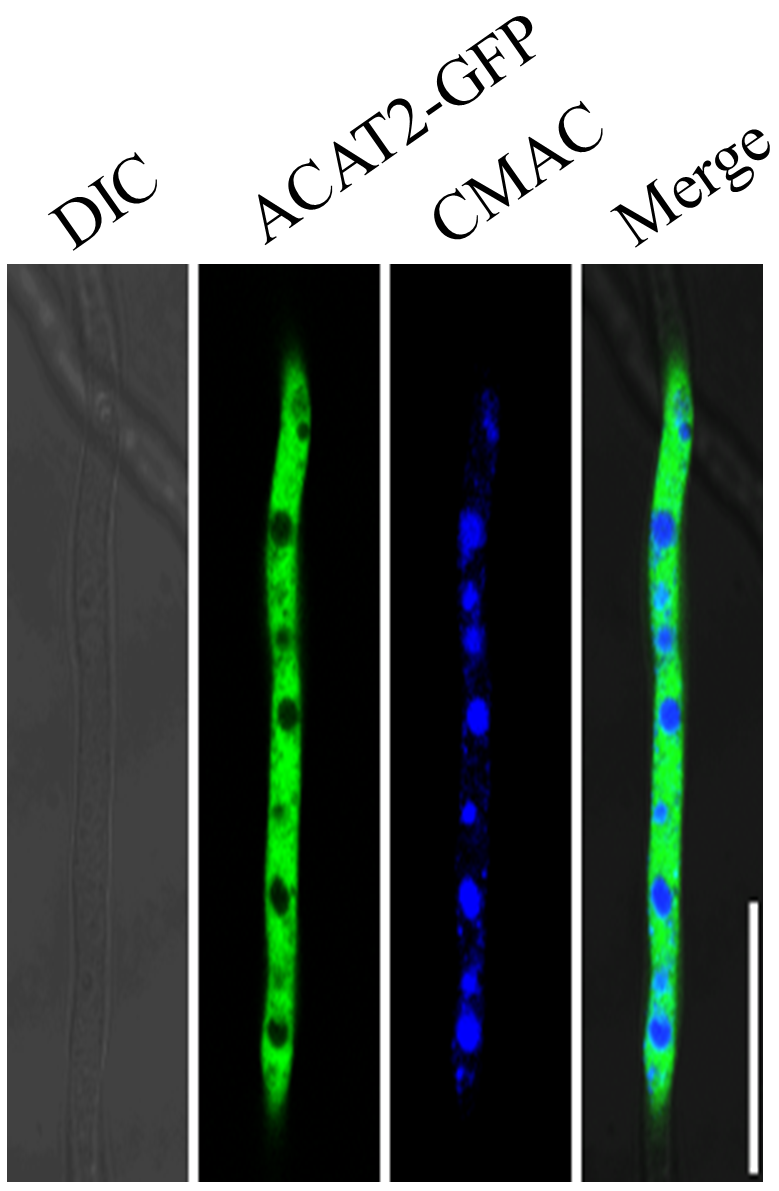

Supplement: Supplementary file 1 — FIGURE S1 FgACAT2 is not localized to the vacuole. Mycelia of FgACAT2 were grown in YEPD and stained with 7‐amino‐4‐chloromethylcoumarin. Bar = 10 μm [file MPP-22-163-s001.tif]

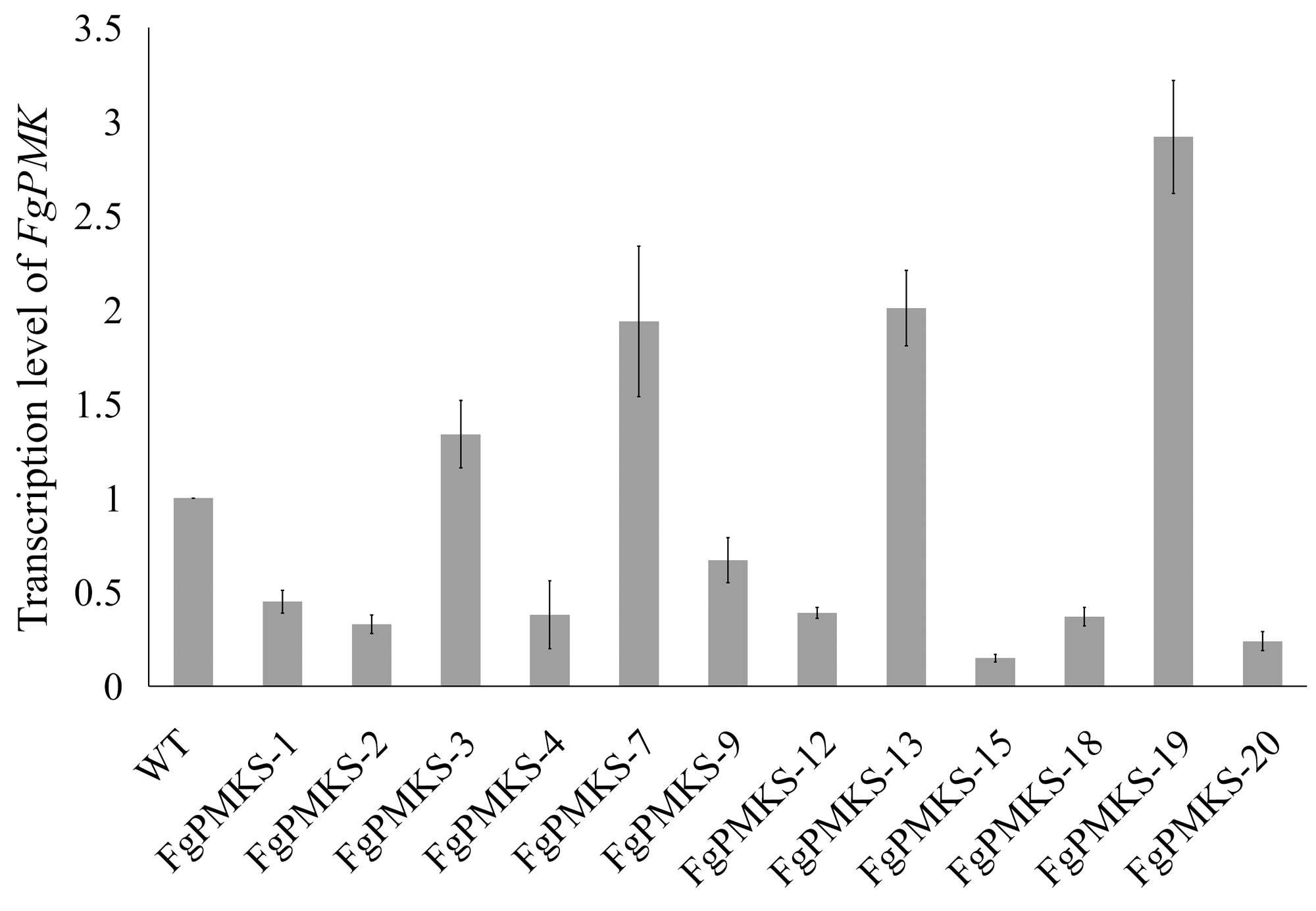

Supplement: Supplementary file 3 — FIGURE S3 Transcription level of FgPMK in different FgPMK silencing transformants [file MPP-22-163-s003.tif]

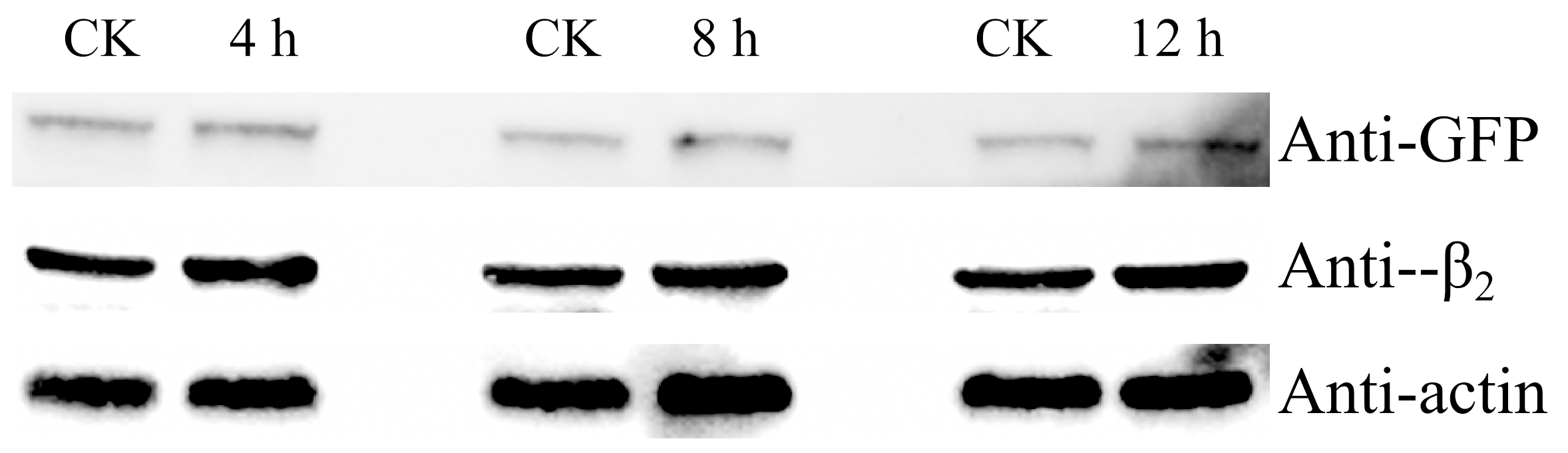

Supplement: Supplementary file 4 — FIGURE S4 Time course analysis of expression of Tri1‐GFP in toxin‐inducing conditions after carbendazim treatment [file MPP-22-163-s004.tif]

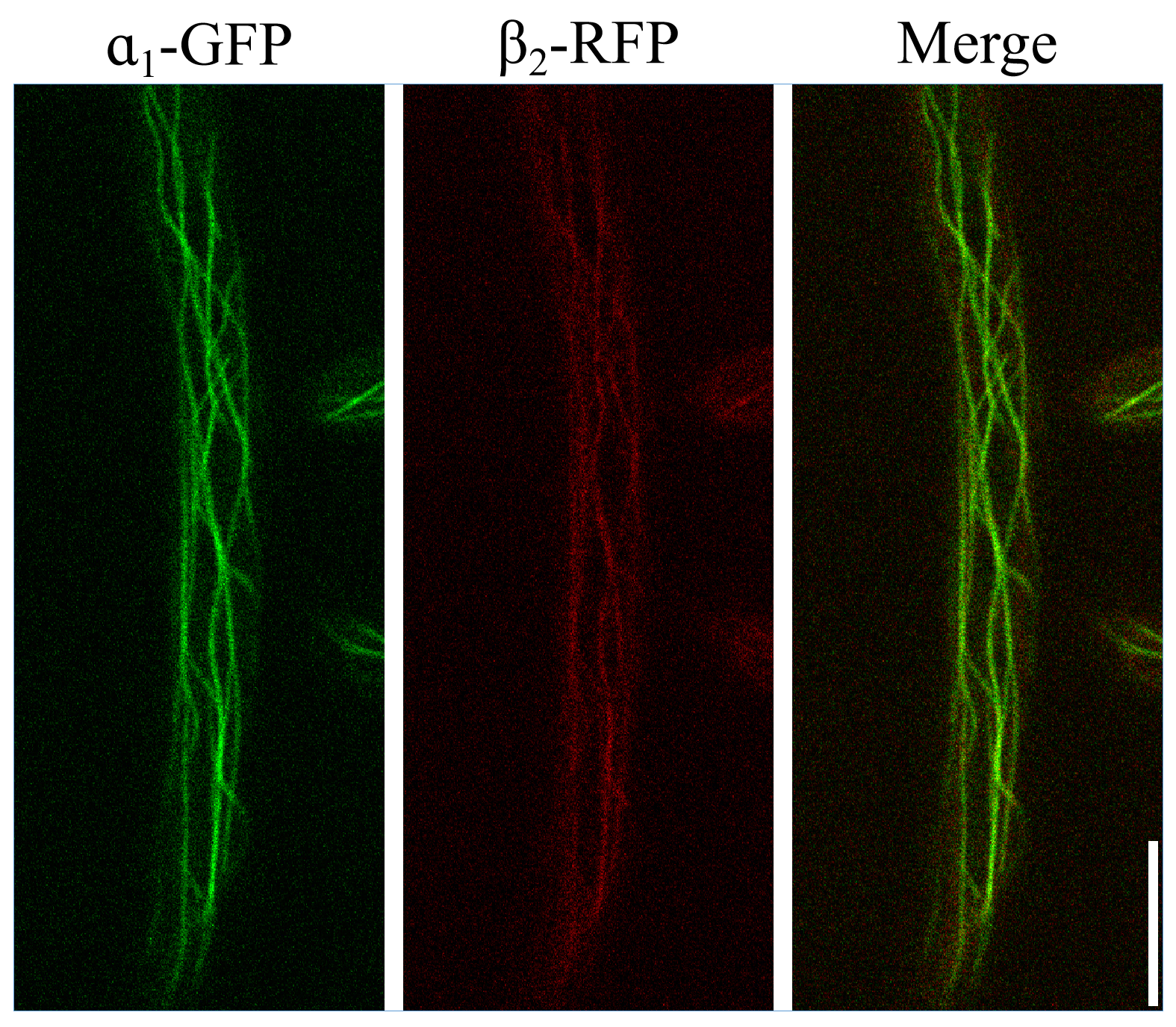

Supplement: Supplementary file 5 — FIGURE S5 Fgα1‐GFP was colocalized with Fgβ2‐RFP in the hyphae of Fusarium graminearum. Bar = 10 μm [file MPP-22-163-s005.tif]

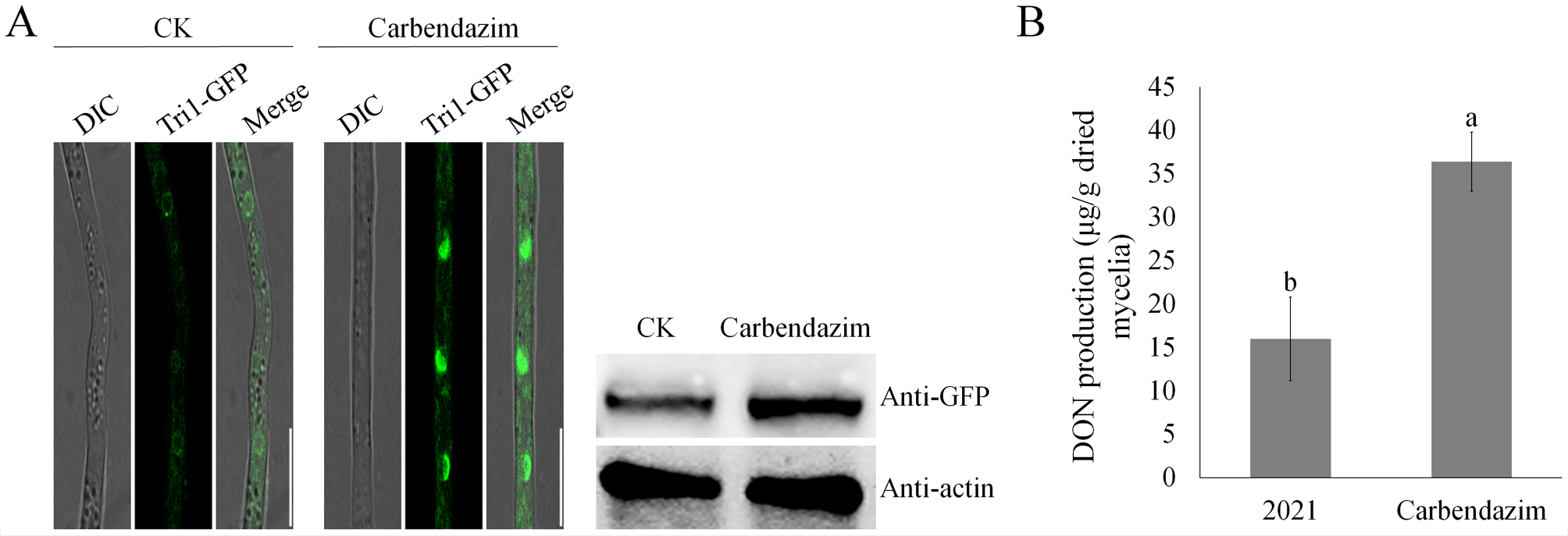

Supplement: Supplementary file 6 — FIGURE S6 The effect of carbendazim treatment (0.5 μg/ml) on DON accumulation. (a) Effects of carbendazim on toxisome assembly. After growth in TBI medium for 24 hr, 0.5 μg/ml carbendazim was added and incubated for another 24 hr. Bar = 10 μm. (b) Effects of carbendazim (0.5 μg/ml) on DON production of hyphae grown in TBI medium. Values on the bars followed by the same letter are not significantly different at α = 0.05 according to Fisher’s LSD test [file MPP-22-163-s006.tif]
