# Supplementary material for: Microtubule‐assisted mechanism for toxisome assembly in Fusarium graminearum
Source: Mol Plant Pathol. 2020 Nov 17;22(2):163–74. doi: 10.1111/mpp.13015 (PMC7814972; doi:10.1111/mpp.13015)
Supplement: Supplementary file 7 — TABLE S1 Wild‐type and mutants of Fusarium graminearum used in this study [file MPP-22-163-s007.doc]

Table S1. **Wild-type and mutant strains of *F. graminearum* used in this study.**

| Strain | Genotype description | Reference |
| --- | --- | --- |
| PH-1 | Wild-type strain of *F. graminearum* | In this lab |
| ∆Fgβ_2_ | Fgβ_2_-tubulin deletion mutant of PH-1 | Qiu *et al.*, 2012 |
| ∆Fgβ_1_ | Fgβ_1_-tubulin deletion mutant of PH-1 | Qiu *et al.*, 2012 |
| ∆Fgɑ_2_ | Fgɑ_2_-tubulin deletion mutant of PH-1 | Hu *et al.*, 2015 and this study |
| ∆Fgɑ_1_ | Fgɑ_1_-tubulin deletion mutant of PH-1 | Hu *et al.*, 2015 and this study |
| PH-1::Tri1-GFP | *FgTri1*-GFP transformant of PH-1 | This study |
| ∆Fgβ_2_::Tri1-GFP | *FgTri1*-GFP transformant of ∆Fgβ_2_ | This study |
| ∆Fgβ_1_::Tri1-GFP | *FgTri1*-GFP transformant of ∆Fgβ_1_ | This study |
| ∆Fgɑ_2_::Tri1-GFP | *FgTri1*-GFP transformant of ∆Fgɑ_2_ | This study |
| ∆Fgɑ_1_::Tri1-GFP | *FgTri1*-GFP transformant of ∆Fgɑ_1_ | This study |
| Fgβ_2_-RFP | *Fgβ_2_*-RFP transformant of ∆Fgβ_2_ | This study |
| Fgɑ_1_-GFP::β_2_ -RFP | *Fgɑ_1_*-GFP transformant of *Fgβ_2_*-RFP | This study |
| Fgβ_1_-RFP::Tri1-  GFP | *Fgβ_1_*-RFP transformant of PH-1::*Tri1*-GFP | This study |
| Fgɑ_1_-3×Flag | *Fgɑ_1_*-3×Flag transformant of ∆Fgɑ_1_ | This study |
| Fgɑ_1_-3×Flag::Tri1-GFP | *FgTri1*-GFP transformant of Fgɑ_1_-3×Flag | This study |
| PH-1::ACAT1-GFP | *FgACAT1*-GFP transformant of PH-1 | This study |
| PH-1::ACAT2-GFP | *FgACAT2*-GFP transformant of PH-1 | This study |
| PH-1::PMK-GFP | *FgPMK*-GFP transformant of PH-1 | This study |
| PH-1::MDV-GFP | *FgMDV*-GFP transformant of PH-1 | This study |
| Tri1-RFP::PMK-  GFP | *Tri1*-RFP transformant of PH-1::PMK-GFP | This study |
| Tri1-RFP::ACAT2-  GFP | *Tri1*-RFP transformant of PH-1::ACAT2-GFP | This study |
| FgPMK-S15 | FgPMK silencing mutant of PH-1 | This study |
| FgPMK-S15::Tri1-GFP | *FgTri1*-GFP transformant of FgPMK-S15 | This study |
