# Supplementary material for: Microtubule‐assisted mechanism for toxisome assembly in Fusarium graminearum
Source: Mol Plant Pathol. 2020 Nov 17;22(2):163–74. doi: 10.1111/mpp.13015 (PMC7814972; doi:10.1111/mpp.13015)
Supplement: Supplementary file 8 — TABLE S2 Primers used in this study [file MPP-22-163-s008.doc]

Table S2. **Primers used in this study.**

| Primer | Sequence (5’-3’) | Application |
| --- | --- | --- |
| GFP-*FgTri1*-1F | ACTCACTATAGGGCGAATTGGGTACTCAAATTGGTTTTGTGAGTAGGCCTCATA | For GFP-FgTri1 fusion construct, native promoter |
| GFP-*FgTri1*-2R | CACCACCCCGGTGAACAGCTCCTCGCCCTTGCTCACGTCATCCTGTACCAATTCCA |
| GFP-*FgACAT1*-1F | ACTCACTATAGGGCGAATTGGGTACTCAAATTGGTTCTATGATGCTGGGCGAAGG | For GFP-FgACAT1 fusion construct, native promoter |
| GFP-*FgACAT1*-2R | CACCACCCCGGTGAACAGCTCCTCGCCCTTGCTCACTACGGACTCAACACGCTGCA |
| GFP-*FgACAT2*-1F | ACTCACTATAGGGCGAATTGGGTACTCAAATTGGTTCCCTTTTCGGGGCTTATTC | For GFP-FgACAT2 fusion construct, native promoter |
| GFP-*FgACAT2*-2R | CACCACCCCGGTGAACAGCTCCTCGCCCTTGCTCACTTGCAGGTTCTCAATGACCA |
| GFP-*FgPMK*-1F | ACTCACTATAGGGCGAATTGGGTACTCAAATTGGTTTGACTCCTTACATCGCCAACA | For GFP-FgPMK fusion construct, native promoter |
| GFP-*FgPMK*-2R | CACCACCCCGGTGAACAGCTCCTCGCCCTTGCTCACGACCCAACCAGCGTACACGT |
| GFP-*FgMDV*-1F | ACTCACTATAGGGCGAATTGGGTACTCAAATTGGTTTTCACTCTGTTCTTCCCGTCT | For GFP-FgMDV fusion construct, native promoter |
| GFP-*FgMDV*-2R | CACCACCCCGGTGAACAGCTCCTCGCCCTTGCTCACAGCGAGGAACTCGTCGGTCT |
| GFP-*Fgɑ_1_*-1F | ACTCACTATAGGGCGAATTGGGTACTCAAATTGGTTCTGGCACTGTCGGTATTTAT | For GFP-Fgɑ_1_ fusion construct, native promoter |
| GFP-*Fgɑ_1_*-1R | CACCACCCCGGTGAACAGCTCCTCGCCCTTGCTCACGTACTCAGCCTCCAACTCCTC |
| RFP-F | ATGGCCTCCTCCGAGGACGTCAT | For RFP fragment construct |
| RFP-R | TTAGGCGCCGGTGGAGTGGC |
| Hph-F | GCCACTCCACCGGCGCCTAAGGGAGCTGTTGGCTGGCTGGT | For Hph fragment construct |
| Hph-R | GGGGAGAGGCGGTTTGCGTATT |
| RFP-*FgTri1*-1F | GAACGCCGTCTTTCTTGTC | For Tri1-RFP-Hph fusion construct, native promoter |
| RFP-*FgTri1*-1R | ATGACGTCCTCGGAGGAGGCCATGTCATCCTGTACCAATTCCA |
| RFP-*FgTri1*-2F | AATACGCAAACCGCCTCTCCCCTTCAACATGGGAATATCGAA |
| RFP-*FgTri1*-2R | CGAACGGCATTGTTAGGTC |
| RFP-*Fgβ_1_*-1F | AGTTTTGGGTCGTGTTTGG | For Fgβ_1_-RFP-Hph fusion construct, native promoter |
| RFP-*Fgβ_1_*-1R | ATGACGTCCTCGGAGGAGGCCATCTCCTCGCCCTCAGGCAGCT |
| RFP-*Fgβ_1_*-2F | AATACGCAAACCGCCTCTCCCCAATTCTAAATTCCTAAACGA |
| RFP-*Fgβ_1_*-2R | GCTGCCCTAACCTCCCTGT |
| 3×Flag-Fgɑ_1_-1F | CTGGCACTGTCGGTATTTAT | For Fgɑ_1_-3×Flag fusion construct, native promoter |
| 3×Flag-Fgɑ_1_-1R | CTTATCGTCGTCATCCTTGTAATCGTACTCAGCCTCCAACTCCTC |
| 3×Flag-Fgɑ_1_-2R | CTTATCGTCGTCATCCTTGTAATCCTTATCGTCGTCATCCTTGTAATCGTACTCAG |
| 3×Flag-Fgɑ_1_-3R | TTACTTATCGTCGTCATCCTTGTAATCCTTATCGTCGTCATCCTTGTAATCCTTATC |
| 3×Flag-Fgɑ_1_-2F | GATTACAAGGATGACGACGATAAGTAATTGTCTGCTTGTTACCAGATG |
| 3×Flag-Fgɑ_1_-4R | AGACCACCAACGCTGCTAT |
| RFP-*Fgβ_2_*-1F | CAATGTTCCAGGGGCGTAG | For Fgβ_1_-RFP fusion construct, native promoter |
| RFP-*Fgβ_2_*-1R | ATGACGTCCTCGGAGGAGGCCATACCCTCGTACTCCTCGGGCT |
| RFP-*Fgβ_2_*-2F | GCCACTCCACCGGCGCCTAA  GATTCTTCCCAAAAGCGGTTTCC |
| RFP-*Fgβ_2_*-2R | GGACGATGGTCTCCGTGTAT |
| pSilent-PMK-1F | CCGCTCGAGCTGGCGGGTATCTCGTCTT | For FgPMK silencing plasmid construct |
| pSilent-PMK-1RF | CCCAAGCTTAAGGAGCGAGGCGGTTAGT |
| pSilent-PMK-2F | GGGGTACCCTGGCGGGTATCTCGTCTT |
| pSilent-PMK-2R | GAAGATCTAAGGAGCGAGGCGGTTAGT |
| Q-PMK-F | CGACTCTTAGCTATGCTCTCAC | For qPCR assay |
| Q-PMK-R | TGTCCGCAAGGATGATAAGG |
